# Retraction: miR-802 participates in the inflammatory process of inflammatory bowel disease by suppressing SOCS5

**DOI:** 10.1042/BSR-2019-2257_RET

**Published:** 2024-11-06

**Keywords:** inflammatory bowel disease, mice colitis model, miR-802, SOCS5, TH17, TNF-α

This Retraction follows an Expression of Concern relating to this article previously published by Portland Press.

This article “miR-802 participates in the inflammatory process of inflammatory bowel disease by suppressing SOCS5” DOI: 10.1042/BSR20192257 is being retracted from *Bioscience Reports* at the request of the authors following receipt of a notification from a reader, alerting the Editorial Office to multiple concerns for the data presented in this article. Specifically:

- Figure 2C inhibitor NC panel contains several repeated patterns in the data points
- Figure 5C which seems to appear in Figure 2E from Li et al, 2020 (DOI: 10.1042/bsr20194427) [retracted]
- Figure 5C GAPDH panel which seems to appear as Figure 3E β-actin panel from Shi et al, 2020 (DOI: 10.2147/OTT.S248797) with horizontal flip transformation

The authors wish to retract the article. The Editor-in-Chief and Editorial Board agree with the retraction.

